# The ‘Sphere’: A Dedicated Bifurcation Aneurysm Flow-Diverter Device

**DOI:** 10.1007/s13239-014-0188-4

**Published:** 2014-08-26

**Authors:** Thomas Peach, J. Frederick Cornhill, Anh Nguyen, Howard Riina, Yiannis Ventikos

**Affiliations:** 1 Institute of Biomedical Engineering, Department of Engineering Science, Oxford University, Oxford, UK; 2 Minimally Invasive New Technologies Program, Weill-Cornell Medical College, New York, NY USA; 3 New York Presbyterian Hospital, New York, NY USA; 4 Department of Neurosurgery, NYU Langone Medical Center, New York, NY USA; 5 Department of Mechanical Engineering, University College London, London, UK

**Keywords:** Minimally invasive, Neurovascular, Thrombosis, Stent, CFD, Medical devices, SILK, PED, WEB, WSS, Shear stress

## Abstract

We present flow-based results from the early stage design cycle, based on computational modeling, of a prototype flow-diverter device, known as the ‘Sphere’, intended to treat bifurcation aneurysms of the cerebral vasculature. The device is available in a range of diameters and geometries and is constructed from a single loop of NITINOL^®^ wire. The ‘Sphere’ reduces aneurysm inflow by means of a high-density, patterned, elliptical surface that partially occludes the aneurysm neck. The device is secured in the healthy parent vessel by two armatures in the shape of open loops, resulting in negligible disruption of parent or daughter vessel flow. The device is virtually deployed in six anatomically accurate bifurcation aneurysms: three located at the Basilar tip and three located at the terminus bifurcation of the Internal Carotid artery (at the meeting of the middle cerebral and anterior cerebral arteries). Both steady state and transient flow simulations reveal that the device presents with a range of aneurysm inflow reductions, with mean flow reductions falling in the range of 30.6–71.8% across the different geometries. A significant difference is noted between steady state and transient simulations in one geometry, where a zone of flow recirculation is not captured in the steady state simulation. Across all six aneurysms, the device reduces the WSS magnitude within the aneurysm sac, resulting in a hemodynamic environment closer to that of a healthy vessel. We conclude from extensive CFD analysis that the ‘Sphere’ device offers very significant levels of flow reduction in a number of anatomically accurate aneurysm sizes and locations, with many advantages compared to current clinical cylindrical flow-diverter designs. Analysis of the device’s mechanical properties and deployability will follow in future publications.

## Introduction

The frequency of intracranial aneurysms in the general population is considered to be between 1 and 5%.^47^ In the vast majority of cases, these aneurysms are asymptomatic, posing no health risk to the patient. In some cases, an aneurysm will rupture, causing a potentially life-threatening hemorrhage. The risk of rupture can be dependent on aneurysm size and geometry but is typically only a few percent for small aneurysms, increasing to up to 50% for giant (>25 mm diameter) lesions.^49^ Intracranial aneurysm rupture is a major cause of stroke, resulting in hemorrhagic stroke or subarachnoid hemorrhage. Aneurysm rupture can lead to significant brain damage and even death in around 25% of hemorrhage cases.^8^ As diagnostic imaging use becomes more widespread, an increasing number of asymptomatic aneurysms are being identified during imaging scans (CT, MRI) requested for other conditions.^47^ This has lead to marked increase in the need for effective treatment options to stabilize the ever-increasing number of aneurysms identified as being at risk of rupture or complications. Treatments focus on reducing the rupture risk by excluding the aneurysm from the circulation, which induces stable thrombus formation within the aneurysm sac, and ultimately, vascular remodeling.

**Figure 1:**
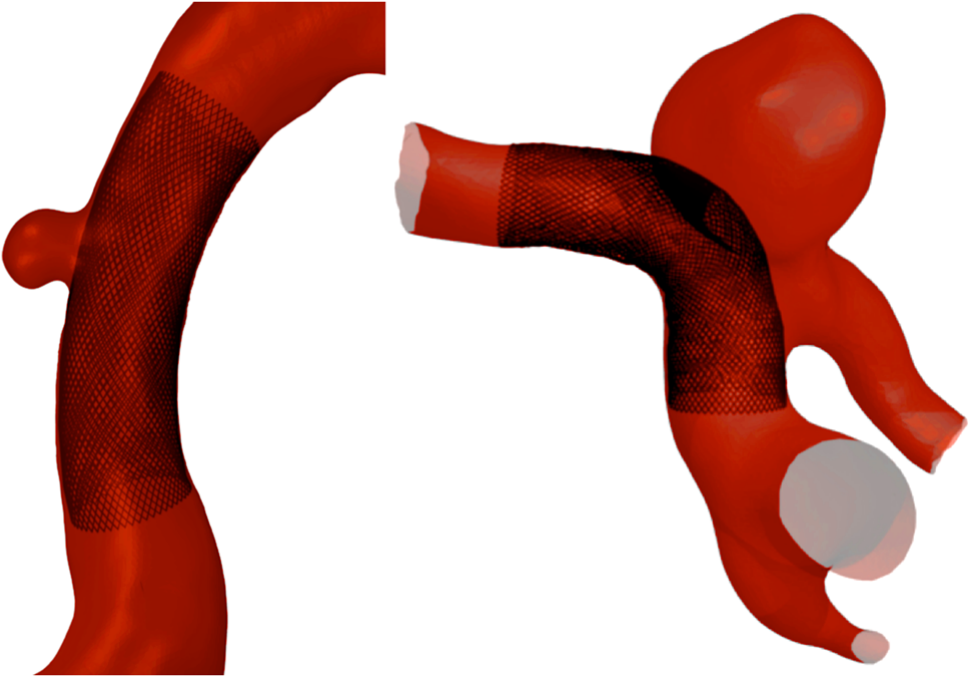
Conventional cylindrical FD treatment of an internal carotid artery (ICA) sidewall aneurysm (left) and the same FD deployed to treat an ICA bifurcation aneurysm, resulting in the occlusion of one daughter vessel (right)

Flow-diverters (FDs) were first proposed in the late 1990s^15^ as a potential treatment for cerebral aneurysms, which would potentially eliminate the problems associated with the placement of coils (e.g., coil migration, mass effect, puncture of aneurysm dome, *etc.*) and achieve aneurysm stabilization by decreasing the blood flow into the aneurysm sac. Flow-diverter treatment aims to reconstruct the parent vessel with a low porosity stent that reduces intra-aneurysmal flow, leading to stable thrombus formation in addition to allowing neointimal and endothelial growth on the stent surface.^30^ A typical flow-diverter of a cylindrical design, placed to treat a sidewall aneurysm, is shown in Fig. 1 (left). A similar design of flow-diverter was first used in a patient in 2006,^25^ and from early experiences of the devices’ usage on a variety of clinical cases, the outcomes have been positive with good aneurysm occlusion rates at times of follow up.^5^,^24^,^25^,^27^ Many authors have shown low flow-diverting effects experienced by small side branches and perforators of a vessel after FD deployment, suggesting there is little risk of secondary infarction following treatment in territories with rich collaterals.^21^ However, the result of large vessel occlusion by flow-diverters, especially at bifurcations, remains unknown.

There are currently two cylindrical designs of cerebral flow-diverter on the market that are approved for use in patients: the *PIPELINE EMBOLIZATION DEVICE*, known as the *PED* (Covidien/ev3, Irvine, CA) and the *SILK* flow-diverter (Balt Extrusion, Montmorency, France), with another four or five in various stages of development. Clinical experience of both the *PIPELINE* 24,27 and the *SILK* 21,25 report good aneurysm occlusion in many different sac sizes and geometries. The body of research detailing clinical experience with both designs of flow-diverter continues to grow, with a total of over 800 interventions recorded in the literature.

While such flow-diverter designs present an elegant treatment solution for sidewall aneurysms, treatment of bifurcation aneurysms with FDs remains problematic. The closed-cell cylindrical nature of current FD designs makes the jailing of all but one daughter vessel inevitable during treatment. This is illustrated in Fig. 1 (right). A study conducted by Saatchi *et al.* 36 included 46 ‘uncoilable’ aneurysms that originated at vessel branches. Although successful treatment with PED was reported in 41 cases, in the five remaining cases (10.9%) complete occlusion of the daughter vessel was reported, which resulted in the death of one patient.

Alternative concepts to the conventional cylindrical flow-diverter have been proposed to better address the treatment of bifurcation aneurysms and reduce treatment morbidity and mortality. The most significant alternative is the *WEB* device (Sequent Medical, Aliso Viejo, CA) The *WEB* device is capable of treating a bifurcation aneurysm without daughter vessel occlusion by morphing a NITINOL^®^ mesh, similar to conventional cylindrical FDs, into a collapsible ball that may be deployed within the aneurysm dome.^20^,^32^ The device is then secured within the aneurysm sac itself by expanding to oppose the vessel wall in a similar manner to conventional coiling. Whether or not a relatively high radial force device deployed inside the aneurysm dome alters any risk of aneurysm weakening, enlarging or rupture is unknown, as is the effect of such forces on wide-neck and giant aneurysms in particular.

In this study we propose a novel design for a device with flow-diverting functionality, intended to specifically treat bifurcation aneurysms. The device, known as the ‘Sphere’, is a NITINOL^®^ spherical frame that presents a high-density face to the aneurysm neck in order to reduce inflow. It is secured by ‘legs’ in the form of multiple open hoops that form part of the spherical frame, which are deployed in the parent vessel distal to the aneurysm. Thus, all bifurcation daughter vessels remain uncompromised following treatment, unlike some interventions using conventional cylindrical FDs. In addition, the device is secured in a non-diseased portion of the artery where high radial force, of the order of conventional cylindrical FDs, is not a concern and no portion of the flow-diverter enters the delicate aneurysm dome. We present an analysis of one of the ‘Sphere’ device designs, of which there are several, deployed in six patient-specific, anatomically accurate aneurysm geometries.

## Literature Review

### Flow-Diverter Devices

A broad spectrum of stent designs has been studied using computational fluid dynamics (CFD) modeling in the literature, ranging from the very abstract to designs that are commercially available. From these studies, it is clear that when considering variations in flow-diverter geometry, porosity has been most widely investigated, with the general and intuitive assumption that lower porosity devices inhibit flow to greater degree. It is widely held that a porosity of around 70% is optimal for flow-diverting stents.^9^,^23^,^42^ Exploring beyond the effect of porosity, Lieber *et al.* 22 reported the effect of strut size on intra-aneurysm flow. The study concluded that at a constant porosity, decreasing the filament diameter and therefore increasing the number of struts across the aneurysm neck reduced inflow more. Fu *et al.* 11 considered the role of stent geometry and surface pattern on aneurysm inflow, with strut cross-section (circular, rectangular, and concave) and overall pattern of the stent (zig-zag or helical) being varied. The greatest reduction in inflow and wall shear stress (WSS) was seen with a design having struts with a rectangular cross-section arranged in a zig-zag pattern. In a paper discussing mesh optimisation strategies, Appanaboyina *et al.* 2 modeled stents with both zig-zag and hexagonal cell designs, finding the greatest reduction in inflow from the zig-zag design.

Current commercially available flow-diverters are dominated by the *SILK/SILK* + (Balt Extrusion, Montmorency, France) and *PED* (EV3/Covidien, Irvine, CA). Both devices have a cylindrical shape composed of a rhomboid-shaped mesh that is woven from NITINOL^®^ wires of approximately 30 *μ*m diameter. The mesh is woven from 48 strands of wire resulting in mesh pore diameters in the range of 110–250 *μ*m. The devices are available in a similar range of diameters (typically 2.5–5 mm) and lengths (10–40 mm) resulting in a device porosity of around 70% regardless of device size or manufacturer.

The *WEB/WEBII* device (Sequent Medical, Aliso Viejo, CA) is the only other commercially available flow-diverting device approved for use in Europe, but not the US, to date. The *WEB* resembles a cylindrical flow diverter with both open ends crimped closed to create a small mesh ball that may be expanded inside the aneurysm sac. The device is available in diameters between 5 and 11 mm and is constructed from a braided mesh comprising 108 or 144 wires, depending on the device size. Two layers of mesh are employed, one inside the other, resulting in flow resistance from a total of 216 or 288 wires. The largest mesh pore diameters observed are in the range of 106–181 *μ*m, depending on device size.^31^ The device has a variable porosity across the face intended to fill the aneurysm neck due to the gathering of the woven mesh at the device’s center. Consequently, the device’s porosity varies from 0% at the device center to 78% at the edges.^20^

### Computational Modeling

An important parameter in patient-specific vascular modeling is the Reynolds number (Eq. 1).

1 $$ \text{Re} = \frac{{\rho {\text{ul}}}}{\mu }, $$

where Re is the Reynolds number, *ρ* the fluid density (kg m^−3^), *u* the mean velocity (m s^−1^), *l* the length scale (pipe diameter, m) and *μ* the dynamic viscosity (Pa s)

Blood flow in the small-diameter vessels that feed the brain typically has a Reynolds number that is less than 1000, suggesting little risk of turbulent transition for the surface roughness of such a vessel.^11^,^19^

The pulsatility effects of blood flow were extensively studied by Womersley, who showed that pulsatile pipe flow adopts an increasingly non-parabolic flow profile with increasing flow frequency.^13^,^50^ The tendency for flow to depart from the steady-state profile and adopt a plug profile increases with the Womersley number, defined in (Eq. 2).

2 $$ \alpha = \frac{l}{2}\sqrt {\frac{\omega \rho }{\mu }} , $$

where *α* is the Womersley number, *ρ* the fluid density (kg m^−3^), *ω* the angular frequency of flow oscillations (*ω* = 2π/T, s^−1^), *l* the length scale (pipe diameter, m) and *μ* the dynamic viscosity (Pa s)

This effect is small at a Womersley number typical of the small blood vessels supplying the brain (when *α* ≈ 1.0–2.0). Flow profiles for the ICA and similar sized vessels have been validated with 3D Doppler measurements and show little departure from a parabolic flow distribution.^18^,^33^ Hence, most studies in the literature model the inlet of a vessel geometry as a parabolic velocity profile consistent with steady, pressure driven flow in a pipe (Poiseuille flow).

Typical mesh independent CFD solutions vary in size across the literature due to different percentage levels of required fidelity to the true solution, to the increase in mesh fineness required to capture complicated patient-specific geometries when compared to more idealized models, or due to different mesh requirements and behavior by different solvers—the latter being of particular importance since it depends directly on the nominal accuracy and numerical features of the solution algorithm employed. Idealized models often are reported utilising meshes of 50,000–500,000 elements,^41^ whereas geometries based on patient-specific data sometimes necessitate 3,000,000–10,000,000 + elements for mesh independence.^6^,^16^,^39^ Introducing a low-porosity stent with small mesh openings into such geometries almost invariably results in finer grid requirements for the same level of mesh independence. Stuhne *et al.* 41 concluded that to reduce random noise in the flow and to fully resolve WSS near to stent struts, the diameter of a mesh element should be less than 1/3 of the strut radius.

### Aneurysm Treatment and Risk of Rupture

Many authors have linked a reduction in either aneurysm inflow (*Q*_in_) or a reduction in mean aneurysm sac velocity to stable thrombus formation.^11^,^17^,^22^ Another measurement linked to thrombus formation and frequently given in the literature is the turnover time before and after FD placement (turnover time = aneurysm sac volume/inflow rate), which allows comparisons between aneurysms with different sac geometries.^16^ Finally, Sadasivan *et al.* 37 modeled aneurysm inflow in the two distinct components of diffusion and convection. Under this regime, resident times of the aneurysm inflow may be calculated with dramatic increases in flow residence time associated with thrombus formation. Such a model gives a ‘virtual contrast’ capability to CFD simulations, allowing direct comparison of results with patient angiograms.

Risk of aneurysm rupture is a complex phenomenon, which remains poorly understood. Both elevated and reduced WSS have been linked to aneurysm formation and increased rupture risk.^1^,^12^,^38^ A study by Chen *et al.* 7 suggested that a number of shear-stress-based metrics (WSS, OSI, GON *etc.*) may have a substantial role in aneurysm enlargement or rupture, but the biochemical basis of such hypotheses remains almost entirely unknown.

The WSS distribution seen in an aneurysm is patient-specific but has been found to correlate with aneurysm aspect ratio (height of aneurysm divided by aneurysm neck diameter): studies have linked an aspect ratio greater than 1.6 with an increased risk of rupture, attributed to an elevated WSS in the dome due to the jetting effect of the geometry.^43^ Flow velocity inside the aneurysm, and consequently WSS distribution, is inversely correlated to the square of the aneurysm neck maximum diameter.^38^ Thus, prediction of aneurysm rupture by size, which is commonly held to be the strongest metric of rupture (especially in the clinical setting), supports the high-WSS theory of rupture. Regardless of competing high or low WSS-induced aneurysm growth and rupture theories, it is widely accepted in the literature that a WSS magnitude around 2 Pa is typical of healthy arterial walls that will retain their structure.^26^ Any large variation from a WSS of around 2 Pa can therefore be considered detrimental and may lead to an increased risk of aneurysm growth or rupture.

Pressure changes within an aneurysm and the surrounding vasculature may also influence aneurysm growth and rupture risk. Cebral *et al.* 6 linked an increase in aneurysm pressure and pressure gradient with rupture, following treatment with a flow-diverter in a study comparing CFD simulations and *in vivo* patient data.

## Methodology

### Governing Equations and Solution Procedure

**Figure 2:**
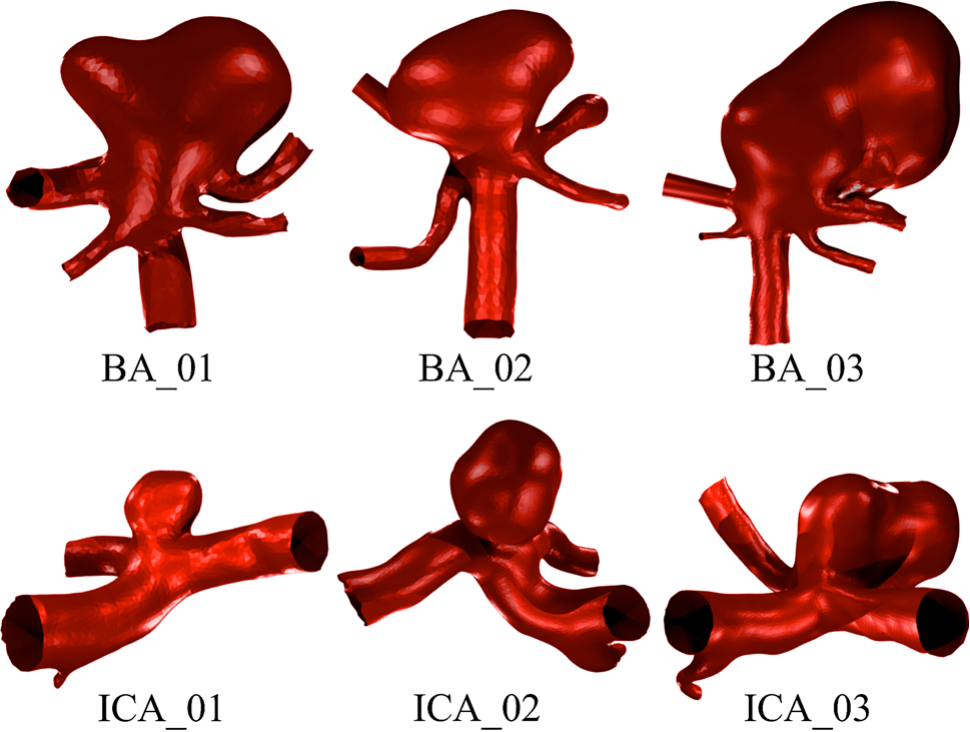
Summary of the six geometries simulated: three Basilar tip aneurysms (Upper: BA_01-03) and three ICA bifurcation aneurysms (Lower: ICA_01-03)

Six anatomically accurate, bifurcation aneurysm geometries are selected: three examples of a Basilar tip aneurysm and three examples of an ICA terminus bifurcation aneurysm. The geometries are segmented from MRA data in *OsiriX* (OsiriX v.4.1.1, Freeware) and converted to STL format before being imported into *Blender* (Stichting Blender Foundation, Amsterdam, The Netherlands). The geometries are trimmed to result in vessel lengths of around five vessel diameters distal and proximal to the aneurysm location, as shown in Fig. 2.

**Figure 3:**
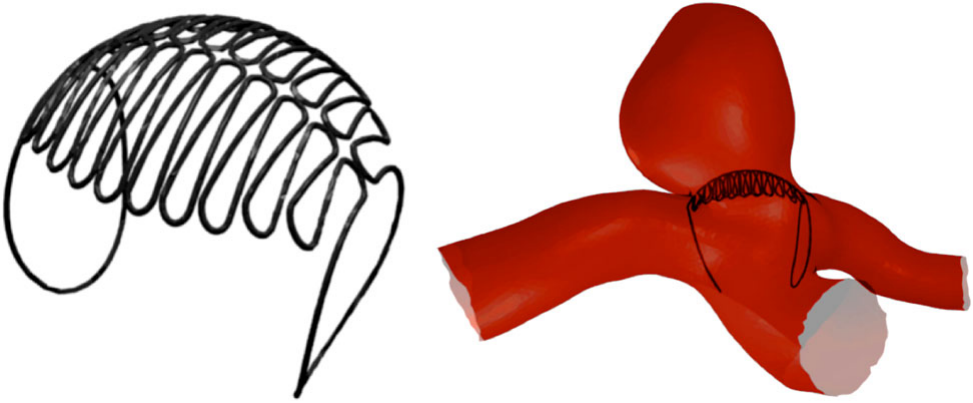
The ‘Sphere’ device in its unconstrained configuration (left) and the typical deployed position in an ICA bifurcation aneurysm (right)

The ‘Sphere’ device is a self-expanding flow-diverter that may be deployed by micro-catheter unsheathing. The device is sized with a diameter between 4.50 and 6.50 mm depending on aneurysm neck size and is constructed from a continuous NITINOL^®^ wire of 102 *μ*m (0.004″) diameter. See Fig. 3 (left). The cap-like dense portion of the device is placed at the aneurysm neck, which is in turn secured by the two device legs that are positioned in the parent vessel proximal to the aneurysm neck. The cap-like portion of the device used in this simulation, which blocks aneurysm inflow, has a porosity of 54%. The ‘Sphere’ has been designed in several configurations with varying geometric designs and face densities. A ‘Sphere’ device is virtually sized to both the aneurysm neck and parent vessel diameters before being virtually deployed. A typical device placement is shown in Fig. 3 (right).

The aneurysm geometries, with and without the device deployed, are imported into *CFD*-*VisCART* (ESI Group, Paris, France) to be meshed. The meshing of each geometry is completed with a Projected Single Domain con-conforming mesh, an Omnitree Cartesian tree type, and three near-wall Cartesian layers to give a smooth and well-resolved boundary definition.

The meshes are imported into the multi physics suite *CFD*-*ACE* + (ESI Group, Paris, France).

Although blood is in general non-Newtonian, it has been shown that the non-Newtonian effects can be assumed secondary in arteries with a diameter greater than 0.5 mm.^29^ Non-Newtonian effects have also been shown to be small inside the aneurysm dome. A negligible difference is seen in flow, pressure and WSS distributions between Newtonian and non-Newtonian models with non-Newtonian models suggesting more stable flow regimes where oscillations are damped by increased viscous forces.^44^ Thus, in this study the blood fluid is assumed incompressible and Newtonian with a density of 1000 kg m^−3^ and a dynamic viscosity of 0.004 Pa s. A rigid arterial wall is assumed, as it has been shown to have little effect on the flow patterns seen when compared to simulations with elastic compliant walls.^10^ Blood flow is modeled as an incompressible fluid with unsteady 3D Navier–Stokes governing equations that are solved following the control (or finite) volume approach, with a Central Differencing scheme for spatial differentiations and interpolations, as well as a Crank–Nicholson second order scheme for time-marching. The SIMPLE-Consistent (SIMPLEC) pressure correction method^28^,^46^ and an algebraic multigrid method for convergence acceleration^48^ are used.

**Figure 4:**
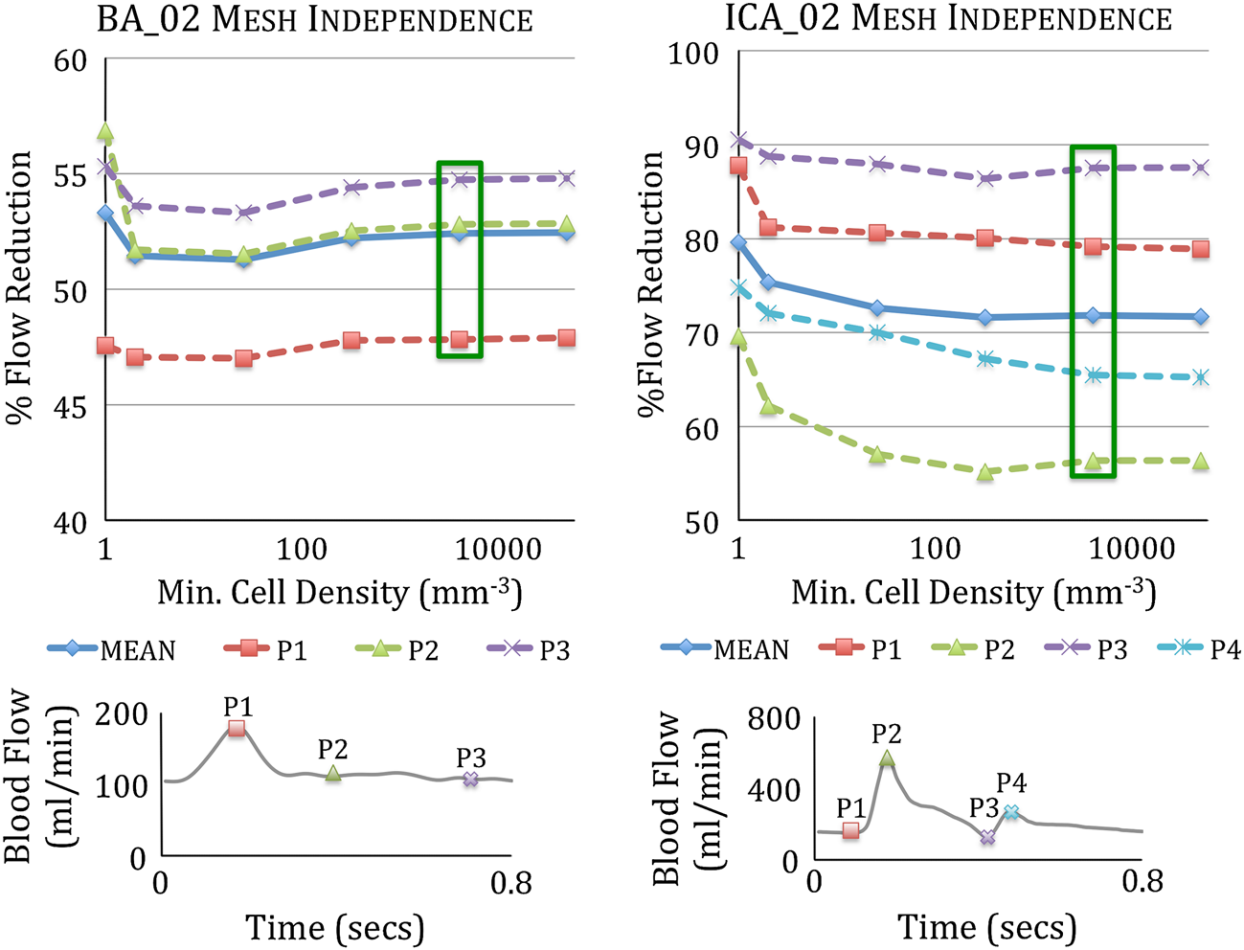
Transient mesh independence test results for geometries ICA_02 and BA_02 plotting percentage inflow reduction due to device deployment with increasing mesh fineness. Both geometries show good convergence of percentage flow reduction in the two finest meshes. The independence criterion of <1% variation in flow reduction between consecutive meshes is met for the mean inflow reduction and salient points in the cardiac cycle (P1, P2 *etc.*) for both geometries at meshes with a cell density greater than ~4000 elements/mm^3^ (indicated in green)

The periodic variation of the mean inlet velocity of each vessel is scaled to fit volumetric flow curves generated from a 1D model of the arterial tree.^34^ Typical flow profiles for the ICA and BA parent vessels over the cardiac cycle are shown in Fig. 4 and have mean flow rates over the cardiac cycle of 230 and 120 mL/min respectively. Steady state tests are run with mean flow of 230 mL/min for the ICA geometries and 120 mL/min for the BA geometries. Poiseuille flow is assumed and a parabolic, radially symmetric inflow profile prescribed. Inlet Reynolds numbers in the range of 274–392 are seen across the steady state simulations of the six geometries.

Transient studies are run with the same meshes but a time-varying inflow based on the profiles of Fig. 4. A radially symmetric inlet velocity profile is prescribed and scaled to result in the same mean flow of the steady state study when averaged over the entire cardiac cycle. This results in transient simulations with mean inlet Reynolds numbers of 274–323 (range of instantaneous Re: 189–403) for the BA vessels and 306–392 (range of instantaneous Re: 169–980) for the ICA vessels. A parabolic inlet velocity profile is used as the relatively small Womersley number of the inflow (1.68–2.72) suggests little departure in velocity profile from a Poiseuille solution.

The option to include outflow boundary conditions derived from either experimentally measured flows or pressures, values originating from 1D models, or simple fixed pressure values was available to the authors. Given that the goal of this study was to compare conditions between untreated and treated vessels, and that access to measurements for the particular aneurysms studied was not available, the simplest possible outlet boundary condition was chosen to avoid spurious assumptions of downstream flow conditions after device deployment. Hence, in both the steady and transient computations an outflow boundary condition of fixed pressure is prescribed at all daughter vessel outlets. Although such a condition may be less realistic than pulsatile 1D analogue models and not fully capture the different back-pressures of the circulatory branches, the relative proportions of outflow in each daughter vessel observed in this study do not differ substantially from *in vivo* results recorded elsewhere in the literature.^14^,^35^

### Mesh Independence

Transient flow mesh independence tests are performed on one aneurysm geometry from each location, with and without a device deployed (in this case geometries BA_02 and ICA_02). The grid independence test metric employed is the mean volumetric flow at the aneurysm neck. Meshes are generated at increasing levels of fineness with minimum cell densities in the range of 1–50,000 elements/mm^3^. The solution is assumed mesh independent when the discrepancy between two consecutive meshes falls below 1% of the total inflow. Aneurysm inflow is measured at salient points in the cardiac cycle (peak systole, dicrotic notch *etc.*) as well as computing a mean aneurysm inflow over a single cardiac cycle. Three full cardiac cycles were simulated with results measured from the final cycle in order to remove transient effects. The study suggests mesh independence at meshes with a typical minimum cell density larger than 4000 elements/mm^3^ as shown in Fig. 4. Additionally, at this level, the number of mesh elements in each geometry measurement plane exceeds the recommendation of Jou *et al.* 17 to fully resolve flow features.

Similar mesh independence tests were performed on all six aneurysm geometries, with and without the device deployed, but for steady-state simulation only. The simulations were run with an inlet velocity typical of the mean velocity seen across the cardiac cycle in the ICA and BA respectively. The steady-state mesh independence test suggested a similar independent cell density (4000 elements/mm^3^) in five of the geometries. No convergent solution was found in the steady state for the ICA_01 geometry, possibly due to intrinsic strong unsteady flow patterns emerging due to flow instabilities, as reported by both Valen-Sendstad *et al.* 45 and Baek *et al.* 3 at high and low frequencies respectively. This extensive mesh independence analysis resulted in mesh size selections that vary between 3.41 and 6.52 million elements across the geometries for both transient and steady state tests and for cases with and without the device implanted.

The independence of the transient flow solution with time-step size was also investigated. Simulations run at a time-step(Δ*t*) of 0.05, 0.01 and 0.005 s respectively indicated that Δ*t* = 0.01 s offered both good convergence of solution, unlike Δ*t* = 0.05 s, and no significant change in flow pattern when compared to Δ*t* = 0.005 s. Such a choice of time-step is consistent with similar CFD studies.^17^,^23^,^45^

Aneurysm inflow is measured through a plane defined at the aneurysm neck. The plane must be placed as close to the natural neck of the aneurysm without falling too close to the device, which can lead to spurious results from local vorticity, as observed by Kim *et al.* 19 For the purpose of our analysis, this plane also defines the boundary between the aneurysm dome and parent vessel.

## Results

Transient simulations are run with a time-step of 0.01 s for three cardiac cycles at 75 bpm, totaling 2.40 s. The simulations are run on up to 32, 2.93 GHz cores with each time-step converging to five orders of magnitude residual reduction in around 50–100 iterations in a typical solution time of 40 min per time step. Steady simulations are run on up to 8, 2.93 GHz cores with a typical solution time around 20 hours to the same residual. No convergent steady-state solution at mean parent vessel flow rate was found for the ICA_01 geometry. An entry plane meeting the criteria previously set out is projected across each aneurysm neck, and the aneurysm inflow at each time step of the transient solution and for the steady state solution is calculated. For both the steady state and transient solutions, the aneurysm inflow before and after device placement are compared to give a percentage flow reduction for a given time step.

**Figure 5:**
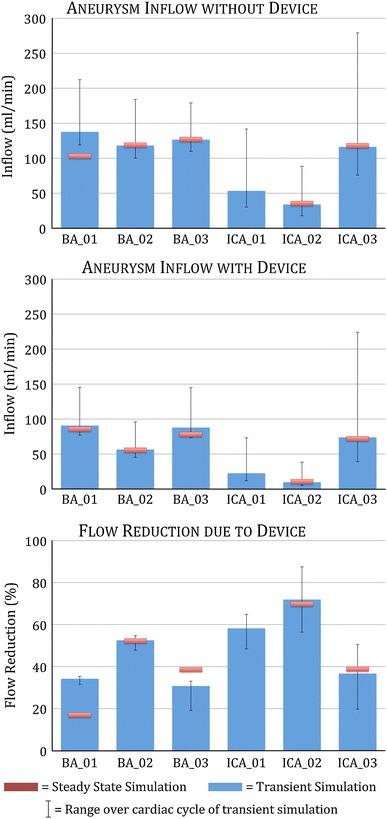
Aneurysm inflow without device (top); aneurysm inflow with device (centre); percentage reduction of aneurysm inflow due to device deployment (bottom) for all six geometries modeled for both steady state and transient blood flow. Results of steady state simulations for flow reduction at mean parent vessel flow are shown in red. The mean and range of flow reduction over the cardiac cycle observed in transient simulations are shown in blue and black respectively

The steady state mean inflow with a device, without a device, and the subsequent flow reduction for each geometry, excluding ICA_01, are plotted in Fig. 5 in red. The mean (over one cardiac cycle) inflow with a device, without a device and the subsequent flow reduction of a transient simulation is found by taking the mean of the flow reduction from each time-step and is plotted in Fig. 5 in blue for each geometry. The range of inflow and flow reduction seen over the cardiac cycle for each geometry is also indicated in Fig. 5, but in black.

The mean flow entering the aneurysm sac in the BA geometries is similar to the mean flow in the parent vessel (120 mL/min), suggesting almost all of the blood flowing in the parent vessel enters the aneurysm. The pattern in the ICA geometries is quite different. Although the mean flow in the ICA geometry parent vessels is almost twice that of the BA cases, at 230 mL/min, the mean flow entering the aneurysm sac in the ICA geometries is equal or substantially less than for the BA cases. The ICA_03 geometry shows a mean aneurysm inflow of approximately half the parent vessel mean flow while the flow entering the aneurysm sac in the ICA_01 and ICA_02 cases is only 5–20% of the parent vessel flow.

A similar pattern is observed after device placement, as shown in the center graph of Fig. 5. Again the aneurysm inflow in the BA geometries exceeds that of the ICA geometries despite the substantially higher parent vessel flow in the ICA cases. Both ICA_01 and ICA_02 also experience a very low level of aneurysm inflow after device placement, which, in the ICA_02 case, corresponds to a mean inflow of less than 5% of the parent vessel flow.

As previously discussed, no convergent solution for the ICA_01 geometry was reached for a steady state simulation of mean parent vessel flow. The steady state simulations of the five other geometries give similar results to the mean result of the corresponding transient simulations with the exception of the BA_01 “no device” and BA_03 “with device” cases. Both the mean and maximum aneurysm inflow with no device predicted by the transient simulation of BA_01 are of note in that they appear non-physical, exceeding the corresponding mean and maximum parent vessel flow rates. An explanation for this phenomenon is discussed in the following section and elucidated in Fig. 8.

**Figure 6:**
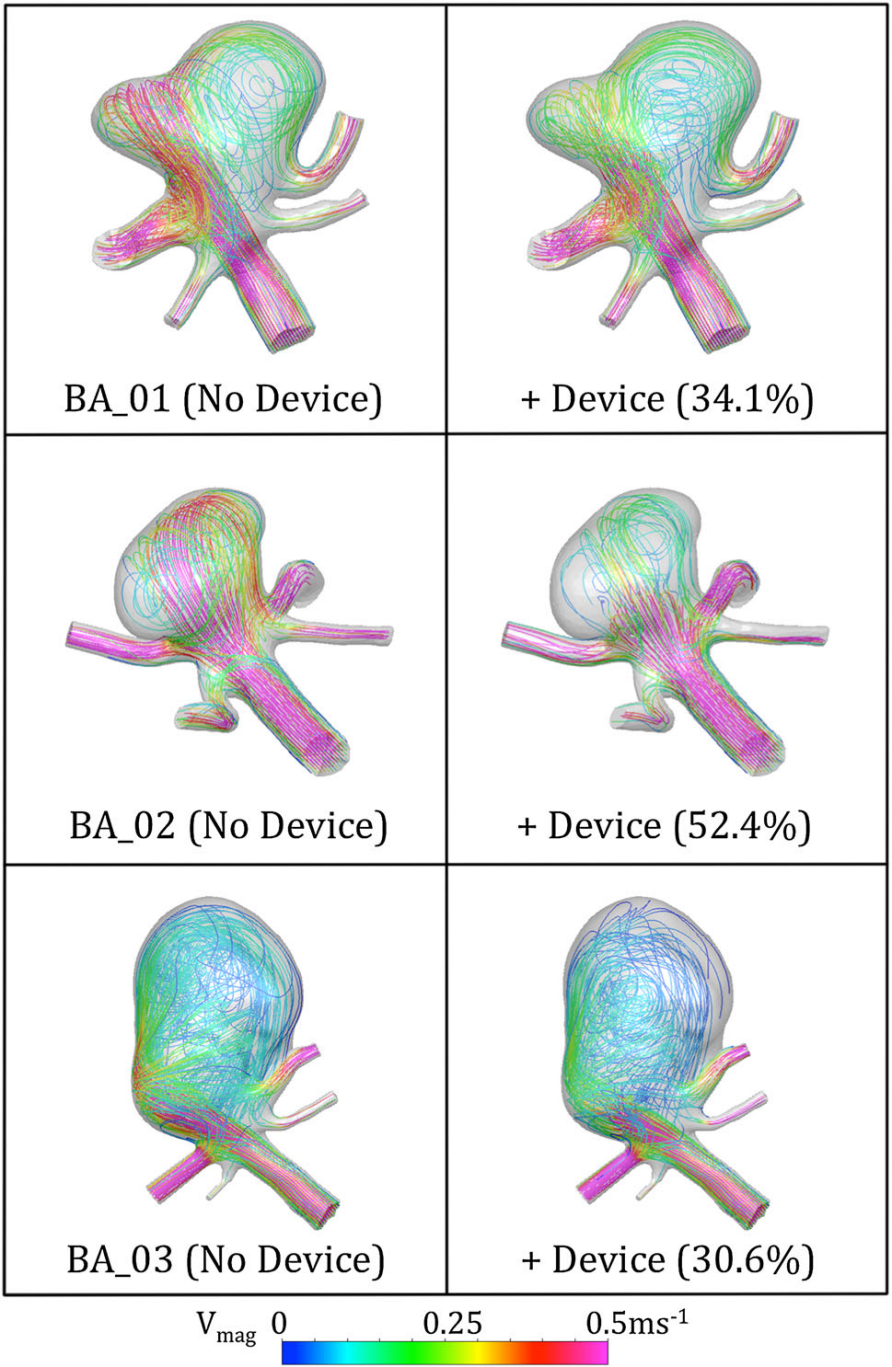
Lines tangent to the instantaneous velocity vectors across all three BA aneurysm geometries with and without a ‘Sphere’ device deployed. The streamlines shown are taken from a time step in the transient solution with mean parent vessel flow. The percentage reduction in aneurysm inflow after device deployment is also indicated for each geometry

Figure 6 shows typical lines tangent to the instantaneous velocity vectors at a point of mean parent vessel flow in each transient simulation for the BA geometries with and without a device deployed. In all three geometries, flow in the aneurysm sac before and after device placement is dominated by a single vortex with a small degree of, possibly, chaotic mixing.^40^ A reduction in this mixing and both the mean and peak flow velocity in the aneurysm sac are seen after device deployment. The BA_02 geometry experiences the greatest reduction in aneurysm inflow and also appears to retain the simplest flow regime (a single vortex with little jetting) both before and after device deployment. The jetting seen in the BA_01 and BA_03 cases with no device is substantially reduced after ‘Sphere’ deployment but the flow reduction seen is some 20% less than for the BA_02 case. A more complex flow pattern remains after device deployment in these two cases with flow violently striking the aneurysm wall before dissipating into a vortex. In all three geometries the device reduces the aneurysm inflow velocity at this mean parent vessel flow condition to typically less than 0.25 ms^−1^.

**Figure 7:**
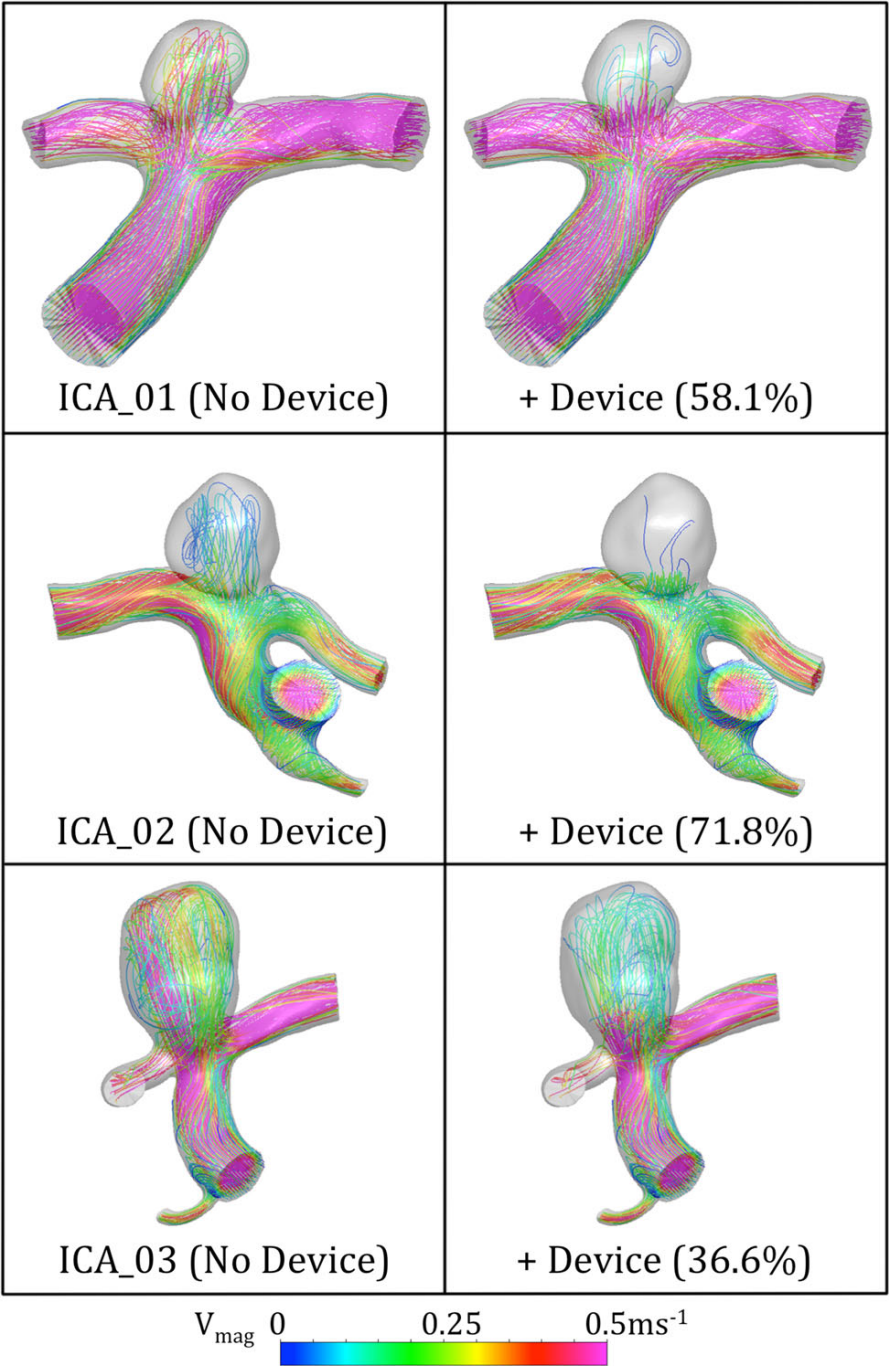
Lines tangent to the instantaneous velocity vectors across all three ICA aneurysm geometries with and without a ‘Sphere’ device deployed. The streamlines shown are taken from a time-step in the transient solution with mean parent vessel flow. The percentage reduction in aneurysm inflow after device deployment is also indicated for each geometry

Figure 7 shows typical lines tangent to the instantaneous velocity vectors for the ICA geometries with and without a device deployed. The mean flow reduction for each geometry is also indicated in both figures. The flow patterns within the aneurysm sac for the ICA cases are also dominated by a single vortex with a small amount of, possibly, chaotic mixing.^40^ The flow patterns appear less complex than for the BA cases but a large variation (almost 40%) in inflow reduction after device deployment is still seen. Flow velocities in the aneurysm sac are greatly reduced after placement of the device but the flow pattern of a single vortex appears unchanged, although this is less clear in the ICA_01 and ICA_02 cases with very low aneurysm inflow after device deployment. The post-device reduction in flow velocity entering the aneurysm sac at mean parent vessel flow is greater than in for the BA cases, with inflow reduced to approximately 0–0.15 ms^−1^.

**Figure 8:**
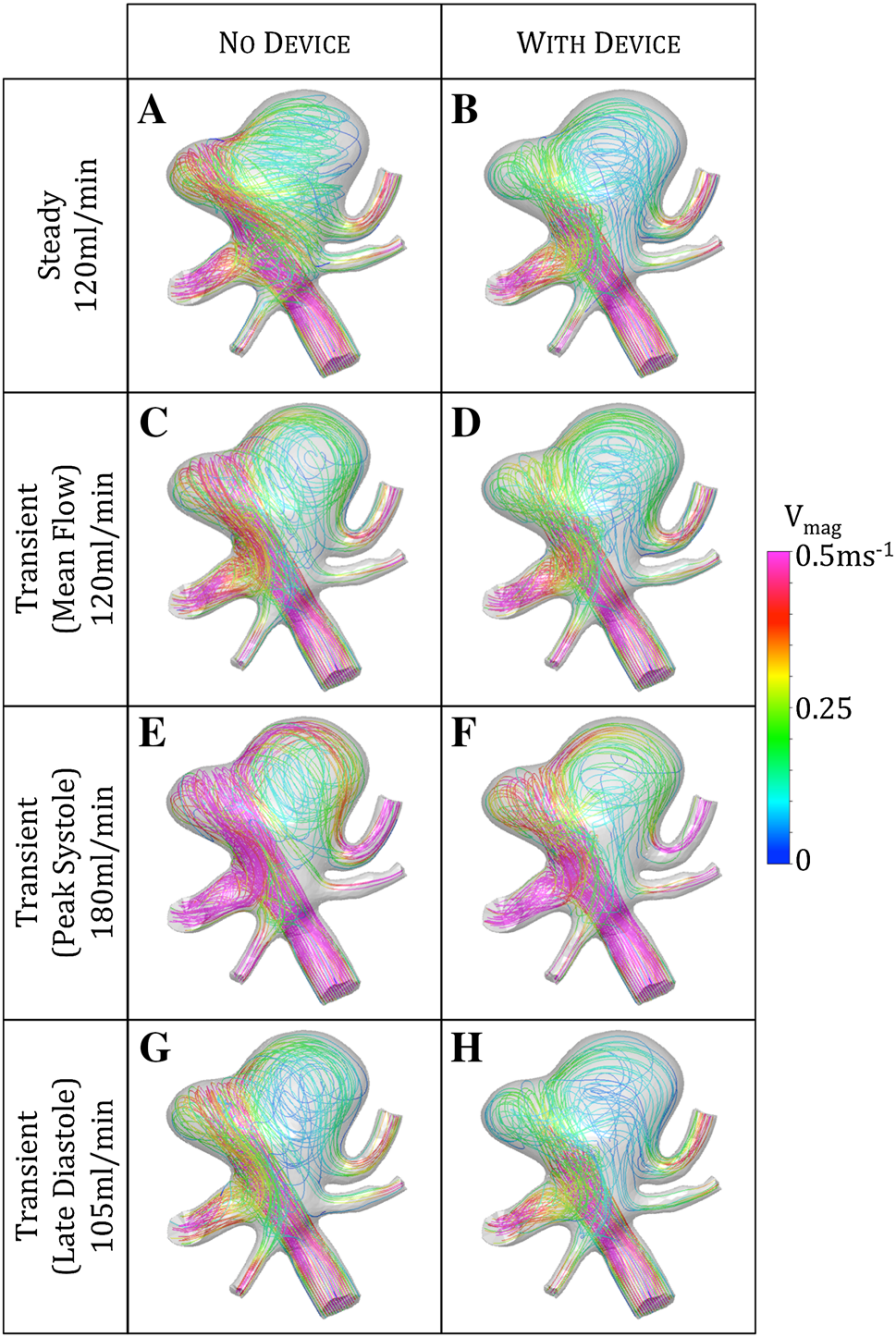
Comparison of steady state (a, b) and transient (c–h) simulations at the same instantaneous parent vessel flow rate of 120 mL/min for the BA_01 geometry with and without the ‘Sphere’ device deployed. A radically different flow pattern is visible in the transient ‘no device’ cases at all points of the cardiac cycle (c, e, g) when compared to the steady prediction (a)

Further detail of the flow regime in the aneurysm sac of the BA_01 geometry for both steady and transient computations is shown in Fig. 8, which is discussed at length in the following section. Broadly, a zone of flow recirculation at the aneurysm neck results in aneurysm inflows that exceed the instantaneous parent vessel flow rate.

**Figure 9:**
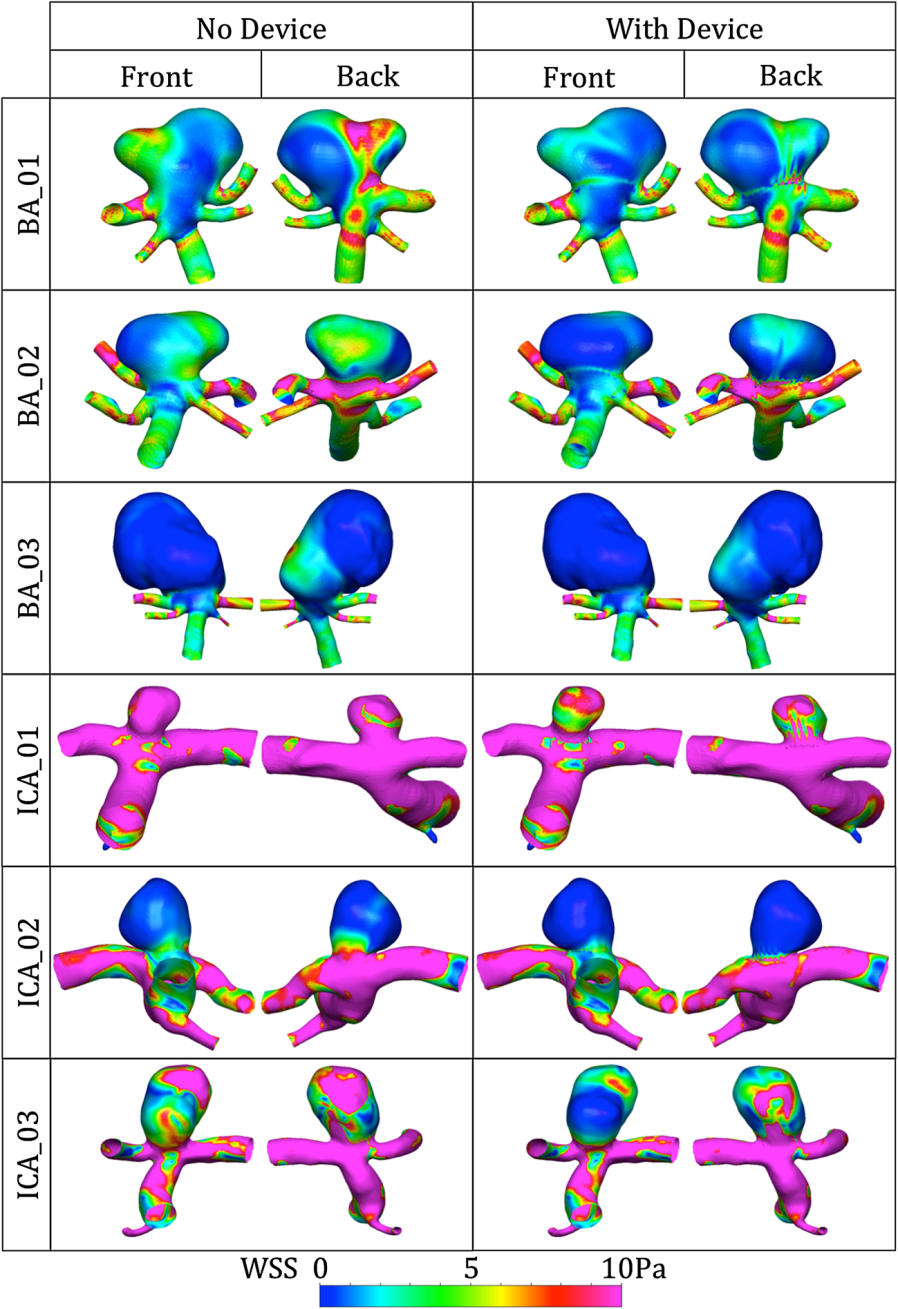
Transient simulation WSS magnitude distributions for each geometry with and without the ‘Sphere’ deployed, viewed from both the front and the back. Results are shown at maximum inflow (peak systole) and although the magnitude of the WSS varies throughout the cardiac cycle, the distribution changes very little from that seen in the figure

Finally, distributions of WSS magnitude are shown in Fig. 9. WSS magnitude is viewed from the front and back of each geometry and both with and without the device deployed. WSS plots are shown for maximum inflow at peak systole, and although the magnitude of the WSS varies, the WSS distribution remains approximately the same across the cardiac cycle. Across all geometries the ‘Sphere’ device reduces the overall WSS magnitude within the aneurysm dome. Peaks in WSS magnitude correspond to areas of flow impingement or jets with high velocity fields near the wall (see Figs. 6, 7) and the reduction in flow jetting after device deployment is also apparent in the WSS distributions and is especially pronounced across the Basilar geometries and in ICA_03.

## Discussion

From the aneurysm inflow with ‘no device’ shown in the first graph of Fig. 5, it is clear that the BA and ICA geometries behave very differently with a much higher aneurysm inflow rate in the BA cases despite the lower parent vessel flow rate of the basilar artery. This phenomenon may be due to the anatomical differences in the BA and ICA termination bifurcations; in the BA cases each aneurysm is relatively wide-necked, with an aneurysm neck diameter larger than the parent vessel diameter, whereas the ICA cases are relatively narrow-necked and typically have an aneurysm neck smaller than the parent vessel. Consequently, the resistance to flow entering the aneurysm in the BA cases is much lower than in the ICA cases, which may explain the reduced flow entering the aneurysms in the ICA geometries despite a much higher parent vessel flow rate than the BA geometries. This hypothesis is also supported by the higher peak and mean velocity of the flow in the aneurysm sac of the BA geometries, despite similar parent vessel velocities between the two vessel types.

From the transient results shown in Fig. 5 (bottom), it can be seen that the ‘Sphere’ device produces a large variation of flow reduction across the six geometries, with a range of 30.6–71.8% in mean reduction and flow reductions as low as 19.1% and as high as 87.5% seen at the peak systole and the dicrotic notch respectively. Consistently across the geometries the greatest reduction in inflow is observed at the point of lowest parent vessel flow (dicrotic notch) and the least reduction in inflow is observed at the highest parent vessel flow (peak systole). In the BA_01 and BA_03 geometries there is a discrepancy between the mean of the transient prediction of flow reduction (blue) and the steady state flow reduction prediction (red) but otherwise the steady state predictions of mean flow reduction are very accurate for the remaining three geometries for which steady state solutions exist.

In both the BA_01‘without device’ and BA_03 ‘with device’ cases, the steady simulation appears to have underestimated the aneurysm inflow. An explanation for this discrepancy is suggested in Fig. 8. The transient simulation of BA_01 reveals recirculation behavior in the right-hand portion of the aneurysm dome with flow both exiting and re-entering the aneurysm neck. This can be clearly seen when comparing the steady and transient ‘without device’ simulations in Fig. 8: the steady simulation (a) suggests a flow pattern with a large low-velocity vortex, in the right-hand portion of the aneurysm dome, with the axis of rotation coincident with the centerline of the parent vessel, whereas the transient simulation (c, e, g) indicates a similar sized low-velocity vortex in the same approximate location but rotating about a perpendicular axis. The vortex seen in the transient case encourages substantial flow recirculation in the aneurysm with flow briefly exiting on the right-hand side of the aneurysm, before being drawn back into the aneurysm close to the centre of the aneurysm neck. This recirculation appears to be the source of the discrepancy between the steady and transient BA_01 results seen in the ‘without device’ graph of Fig. 4. The discrepancy does not appear as dramatic for the same geometry with the ‘Sphere’ deployed, as the device appears to be reducing the momentum of the aneurysm inflow sufficiently to promote a flow pattern where the vortex is weaker and is rotating about an axis closer to that in the steady state prediction as seen Figs. 8b, 8d, 8f, 8h. This also explains the seemingly non-physical result shown in the BA_01 ‘without device’ case where a mean aneurysm inflow of 137 mL/min is calculated despite a mean parent vessel flow of only 120 mL/min. The additional 17 mL/min results from the recirculation of flow exiting and re-entering the aneurysm neck. The same effect is present in the BA_03‘with device’ geometry but to a lesser extent.

Considering Figs. 6 and 7, it appears the action of the ‘Sphere’ is to reduce the velocity and intensity of the flow entering the aneurysm sac with little change in the general flow pattern within the aneurysm, with the exception of BA_01 as previously discussed. In all geometries with no device deployed, the flow in the aneurysm sac appears to be dominated by a single vortex accompanied by a small degree of chaotic mixing, where the inflow jet first strikes the aneurysm wall, as also observed by Butty *et al.* 4 Such mixing appears less prevalent in the BA_02 and ICA_02 geometries, which both saw flow reduction in excess of 50% after device deployment. The ICA_01 geometry, however, which also saw a substantial flow reduction, has a flow pattern dominated by mixing that was subsequently reduced to a very slow moving single vortex after device deployment.

As previously discussed, the precise mechanisms of high or low WSS-induced aneurysm growth and rupture are still hotly debated; in this case, the ‘Sphere’ device appears to promote a healthier hemodynamic environment within the aneurysm sac by reducing the peaks of the WSS distribution to a more physiologically normal value (~2 Pa) as shown in Fig. 9. The larger ICA flow rate results in increased blood flow velocity and hence higher WSS in the ICA cases. This is particularly noticeable in the ICA_01 geometry, which has a below-average vessel diameter (<3 mm) resulting is very large velocities (Fig. 7) and hence high WSS magnitude throughout the geometry. This effect is likely to be lessened physiologically with more elastic deformation of the larger ICA vessels, in turn reducing the WSS magnitude.

As previously discussed, the geometries that experience the greatest flow reduction after device deployment appear to have less flow impingement/jetting in the aneurysm sac prior to device placement. This is reinforced by the WSS distributions of both the BA_02 and ICA_02 geometries where no high WSS (>5 Pa) regions caused by flow jets occur in the aneurysm dome. Conversely, the three geometries that perform less favorably in terms of device flow reduction (BA_01, BA_03 and ICA_3) also have high WSS distributions with peaks from flow jets in the aneurysm sac before device placement. The ICA_01 case does not fit this pattern of high WSS in the aneurysm sac corresponding to poor flow reduction by the device, but the smaller size of the ICA_01 aneurysm (with a height and width roughly equal to the parent vessel diameter) results in less flow jetting generally and a flow pattern in the aneurysm that is more similar to the BA_02 and ICA_02 geometries. It is also apparent that higher aneurysm inflow, either with or without the device (detailed in Fig. 5), does not appear to correlate with increased WSS magnitude in the aneurysm sac.

The authors also created plots of pressure for each vessel before and after device deployment, which are not included here as no salient changes were seen, with the exception of a small increase in inlet pressure after device deployment, corresponding to the increased resistance to flow in the vessel. The large increases in aneurysm sac pressure correlated with aneurysm rupture, which were observed by Cebral *et al.* 6 following FD deployment, would not be detected in this study, as the pressure increase was attributed to the reversal of parent vessel stenosis, which cannot be modeled when assuming a rigid vessel wall as in the case of the current study.

The correlation between a number of geometric features of each aneurysm and the aneurysm inflow calculated before and after device placement was investigated. No correlation was found with parent or daughter vessel size or with the proportion of outflow leaving each daughter vessel. These factors were also considered for the flow reduction achieved by the device with no correlation found. No correlation was found between the ‘no device’ aneurysm inflow and the subsequent effectiveness of the deployed device. The asymmetric distribution of outflow between daughter vessels across the geometries was found to have no correlation with device effectiveness. The ‘peakedness’ (defined as the ratio of maximum inlet velocity to mean inlet velocity and similar to the kurtosis in this case) of the flow profile entering the aneurysm was also found to have no correlation with the flow reduction due to the device.

In the authors’ experience, the flow reductions from the ‘Sphere’ device deployed in bifurcation aneurysms are comparable to those produced by conventional low-porosity stents such as the *SILK/PED*, for sidewall and bifurcation aneurysms. It is intended that the full details of such a comparative study will be the basis of a future publication and are not included here for brevity. Although such flow reductions are comparable, bifurcation aneurysms present significantly more clinical difficulties than sidewall cases, and the treatment of bifurcation aneurysms with conventional FDs is often seen as a treatment of last resort.^21^ The possibility of using the ‘Sphere’ device to treat such difficult aneurysms, and especially difficult cases that are also wide-necked such as the BA_01 geometry, without the placement of the device within the delicate aneurysm sac is also highly desirable.

The current study has shown the ‘Sphere’ device to have a good flow-diverting ability in a number of bifurcation aneurysm geometries. However, significant aspects of the device’s design have not been analyzed: chiefly the device’s manufacture, deliverability, and the mechanical deformations experienced by both device and vessel wall that govern device security. Of these elements the question of device security and the prevention unintended migration is of paramount importance; the authors are currently confronting this design issue and intend to publish further analysis in future publications.

## Conclusions

Preliminary CFD analysis in a number of aneurysm locations and geometries indicates that the ‘Sphere’ design in this study is a viable flow-diverter design for treating bifurcation aneurysms by introducing substantial aneurysm inflow decrease. A range of flow reductions, which are comparable to those achieved with commercially available devices, is seen across the six aneurysms simulated. In all six geometries the device is found to reduce the WSS distribution within the aneurysm sac to values closer to a healthy vessel. The effectiveness of the ‘Sphere’ device is compared to a number of geometric and flow-based features of each aneurysm geometry, where no correlations have been observed.

## References

[1] Alfano J, Kolgega J, Natarajan SK, Xiang J, Paluch R, Levy E, Siddiqui AH, Meng H (2013). Intracranial aneurysms occur more frequently at bifurcation sites that typically experience higher hemodynamic stresses. Neurosurgery.

[2] Appanaboyina S, Mut F, Putman CM, Lohner R, Cebral JR (2008). Computational fluid dynamics of stented intracranial aneurysms using adaptive embedded unstructured grids. Int. J. Numer. Methods Fluids.

[3] Baek H, Jayaraman MV, Richardson PD, Karniadakis GE (2010). Flow instability and wall shear stress variation in intracranial aneurysms. J. R. Soc. Interface.

[4] Butty VD, Gudjonsson K, Buchel P, Makhijani VB, Ventikos Y, Poulikakos D (2002). Residence times and basins of attraction for a realistic right internal carotid artery with two aneurysms. Biorheology.

[5] Byrne, J. V, and I. Szikora. Flow diverters in the management of intracranial aneurysms: a review. *EJMINT*, 2012.

[6] Cebral JR, Mut F, Raschi M, Scrivano E, Ceratto R, Lylyk P, Putman CM (2011). Aneurysm rupture following treatment with flow-diverting stents: computational hemodynamics analysis of treatment. AJNR.

[7] Chen H, Selimovic A, Thompson H, Chiarini A, Penrose J, Ventikos Y, Watton PN (2013). Investigating the influence of haemodynamic stimuli on intracranial aneurysm inception. Ann. Biomed. Eng..

[8] Connolly ES, Rabinstein AA, Carhuapoma JR, Derdeyn CP, Dion J, Dion J, Higashida RT, Hoh BL, Kirkness CJ, Kirkness CJ, Naidech AM, Ogilvy CS, Patel AB, Thompson BG, Vespa P (2012). Guidelines for the management of aneurysmal subarachnoid hemorrhage: a guideline for healthcare professionals from the American Heart Association/American Stroke Association. Stroke.

[9] D’Urso PI, Lanzino G, Cloft HJ, Kallmes DF (2011). Flow diversion for intracranial aneurysms: a review. Stroke.

[10] Dempere-Marco L, Oubel E, Castro M, Putman C, Frangi A, Cebral J (2006). CFD analysis incorporating the influence of wall motion: application to intracranial aneurysms. MICCAI.

[11] Fu W, Gu Z, Meng X, Chu B, Qiao A (2010). Numerical simulation of hemodynamics in stented internal carotid aneurysm based on patient-specific model. J. Biomech..

[12] Griffith TM (1994). Modulation of blood flow and tissue perfusion by endothelium-derived relaxing factor. Exp. Physiol..

[13] Hale BYJF, Mcdonald DA, Womersley JR (1955). Velocity profiles of oscillating arterial flow, with some calculations of viscous drag and the reynolds number. Physiology.

[14] Hennerici M, Rautenberg W, Sitzer G, Schwartz A (1987). Transcranial Doppler ultrasound for the assessment of intracranial arterial flow velocity—part 1. Surg. Neurol..

[15] Higashida R, Smith W, Gress D, Urwin R, Dowd C, Balousek P, Halbach V (1997). Intravascular stent and endovascular coil placement for a ruptured fusiform aneurysm of the basilar artery. Neurosurgery.

[16] Janiga G, Rössl C, Skalej M, Thévenin D (2013). Realistic virtual intracranial stenting and computational fluid dynamics for treatment analysis. Biomechanics.

[17] Jou L-D, Mawad ME (2011). Hemodynamic effect of neuroform stent on intimal hyperplasia and thrombus formation in a carotid aneurysm. Med. Eng. Phys..

[18] Kamenskiy AV, Dzenis YA, Mactaggart JN, Desyatova AS, Pipinos II (2011). *In vivo* three-dimensional blood velocity profile shapes in the human common, internal, and external carotid arteries. Vasc. Surg..

[19] Kim M, Taulbee D, Tremmel M, Meng H (2009). Comparison of two stents in modifying cerebral aneurysm hemodynamics. Ann. Biomed. Eng..

[20] Klisch J, Sychra V, Strasilla C, Liebig T, Fiorella D (2011). The woven endobridge cerebral aneurysm embolization device (WEB II): initial clinical experience. Neuroradiology.

[21] Kulcsár Z, Ernemann U, Wetzel SG, Bock A, Goericke S, Panagiotopoulos V, Forsting M, Ruefenacht DA, Wanke I (2010). High-profile flow diverter (silk) implantation in the basilar artery. Stroke.

[22] Lieber BB, Livescu V, Hopkins LN, Wakhloo AK (2002). Particle image velocimetry assessment of stent design influence on intra-aneurysmal flow. Ann. Biomed. Eng..

[23] Liou T-M, Li Y-C, Juan W-C (2007). Numerical and experimental studies on pulsatile flow in aneurysms arising laterally from a curved parent vessel at various angles. Biomechanics.

[24] Lylyk P, Miranda C, Ceratto R, Ferrario A, Scrivano E, Luna HR, Berez AL, Tran Q, Nelson PK, Fiorella D (2009). Curative endovascular reconstruction of cerebral aneurysms with the pipeline embolization device. Neurosurgery.

[25] Maimon S, Gonen L, Nossek E, Strauss I, Levite R, Ram Z (2012). Treatment of intra-cranial aneurysms with the SILK flow diverter: 2 years’ experience with 28 patients at a single center. Acta Neurochir. (Wien).

[26] Malek AM, Alper SL, Izumo S (1999). Hemodynamic shear stress and its role in atherosclerosis. J. Am. Med. Assoc..

[27] Nelson PK, Lylyk P, Szikora I, Wetzel SG, Wanke I, Fiorella D (2011). The pipeline embolization device for the intracranial treatment of aneurysms trial. AJNR.

[28] Ni M, Abdou MA (2007). A bridge between projection methods and simple type methods for incompressible navier—Stokes equations. IJNMBE.

[29] Perktold K, Resch M, Florian H (1991). Pulsatile non-Newtonian flow characteristics in a three-dimensional human carotid bifurcation model. Biomech. Eng..

[30] Pierot L (2011). Flow diverter stents in the treatment of intracranial aneurysms: where are we?. Neuroradiology.

[31] Pierot L, Liebig T, Sychra V, Kadziolka K, Dorn F, Strasilla C, Kabbasch C, Klisch J (2012). Intrasaccular flow-disruption treatment of intracranial aneurysms: preliminary results of a multicenter clinical study. AJNR.

[32] Pierot L, Klisch J, Cognard C, Szikora I, Mine B, Kadziolka K, Sychra V, Gubucz I, Januel A-C, Lubicz B (2013). Endovascular WEB flow disruption in middle cerebral artery aneurysms: preliminary feasibility, clinical, and anatomical results in a multicenter study. Neurosurgery.

[33] Ponzini R, Vergara C, Rizzo G, Veneziani A, Roghi A, Vanzulli A, Parodi O, Redaelli A (2010). Womersley number-based estimates of blood flow rate in doppler analysis *in vivo* validation by. IEEE Trans. Biomed. Eng..

[34] Reymond P, Vardoulis O, Stergiopulos N (2012). Generic and patient-specific models of the arterial tree. J. Clin. Monit. Comput..

[35] Ringelstein E, Kahlscheuer B, Niggemeyer E, Otis S (1990). Transcranial doppler sonography: anatomical landmarks and normal velocity values. Ultrasound Med. Biol..

[36] Saatchi I, Yavuz K, Ozer C, Geyik S, Cekirge HS (2012). Treatment of intracranial aneurysms using the pipeline flow-diverter embolization device: a single-center experience with long-term follow-up results. AJNR.

[37] Sadasivan C, Lieber BB, Gounis MJ, Lopes DK, Hopkins LN (2002). Angiographic quantification of contrast medium washout from cerebral aneurysms after stent placement. AJNR. Am. J. Neuroradiol..

[38] Sforza DM, Putman CM, Cebral JR (2009). Hemodynamics of cerebral aneurysms. Annu. Rev. Fluid Mech..

[39] Shobayashi, Y., S. Tateshima, R. Kakizaki, R. Sudo, K. Tanishita, and F. Viñuela. Intra-aneurysmal hemodynamic alterations by a self-expandable intracranial stent and flow diversion stent. *Neurointerventional Surg.* 1–5, 2012. doi:10.1136/neurintsurg-2012-010488. 10.1136/neurintsurg-2012-010488 23048176

[40] Sotiropoulos F, Ventikos Y, Lackey T (2001). Chaotic advection in three-dimensional stationary vortex-breakdown bubbles: Sil’nikov’s Chaos and the Devil’s Staircase. Fluid Mech..

[41] Stuhne GR, Steinman DA (2004). Finite-element modeling of the hemodynamics of stented aneurysms. Biomech. Eng..

[42] Trager A, Sadasivan C, Seong J, Lieber BB (2009). Correlation between angiographic and particle image velocimetry quantification of flow diverters in an *in vitro* model of elastase-induced rabbit aneurysms. Biomed. Eng. (NY).

[43] Ujiie H, Tamano Y, Sasaki K, Hori T (2001). Is the aspect ratio a reliable index for predicting the rupture of a saccular aneurysm?. Neurosurgery.

[44] Valencia AA, Guzmán AM, Finol EA, Amon CH (2006). Blood flow dynamics in saccular aneurysm models of the basilar artery. Biomech. Eng..

[45] Valen-Sendstad K, Mardal KA, Steinman DA (2012). High-resolution computational fluid dynamics detects high-frequency velocity fluctuations in bifurcation, but not sidewall, aneurysms of the middle cerebral artery. J. Biomech..

[46] Van Doormaal JP, Raithby GD (1984). Enhancements of the simple method for predicting incompressible fluid flows. Numer. Heat Transf..

[47] Vernooij MW, Ikram MA, Tanghe HL, Vincent AJPE, Hofman A, Krestin GP, Niessen WJ, Breteler MMB, van der Lugt A (2007). Incidental findings on brain mri in the general population. N. Engl. J. Med..

[48] Webster R (1994). An algebraic multigrid solver for Navier–Stokes problems. Int. J. Numer. Methods Fluids.

[49] Wiebers DO, Whisnant JP, Huston J, Meissner I, Brown RD, Piepgras DG, Forbes GS, Thielen K, Nichols D, O’Fallon WM, Peacock J, Jaeger L, Kassell NF, Kongable-Beckman GL, Torner JC (2003). Unruptured intracranial aneurysms: natural history, clinical outcome, and risks of surgical and endovascular treatment. Lancet.

[50] Womersley JR (1955). Method for the calculation of velocity, rate of flow and viscous drag in arteries when the pressure gradient is known. J. Physiol..

